# Empagliflozin increases kidney weight due to increased cell size in the proximal tubule S3 segment and the collecting duct

**DOI:** 10.3389/fphar.2023.1118358

**Published:** 2023-03-23

**Authors:** Frederick Sinha, Anna Federlein, Annika Biesold, Magdalena Schwarzfischer, Katharina Krieger, Frank Schweda, Philipp Tauber

**Affiliations:** Institute of Physiology, University of Regensburg, Regensburg, Germany

**Keywords:** SGLT2 inhibition, empagliflozin, kidney weight, hypertrophy, HIF1α, pH

## Abstract

The inhibition of renal SGLT2 glucose reabsorption has proven its therapeutic efficacy in chronic kidney disease. SGLT2 inhibitors (SGLTi) have been intensively studied in rodent models to identify the mechanisms of SGLT2i-mediated nephroprotection. So far, the overwhelming effects from clinical trials, could only partially be reproduced in rodent models of renal injury. However, a commonly disregarded observation from these studies, is the increase in kidney weight after SGLT2i administration. Increased kidney mass often relies on tubular growth in response to reabsorption overload during glomerular hyperfiltration. Since SGLT2i suppress hyperfiltration but concomitantly increase renal weight, it seems likely that SGLT2i have a growth promoting effect on the kidney itself, independent of GFR control. This study aimed to investigate the effect of SGLT2i on kidney growth in wildtype animals, to identify enlarged nephron segments and classify the size increase as hypertrophic/hyperplastic growth or cell swelling. SGLT2i empagliflozin increased kidney weight in wildtype mice by 13% compared to controls, while bodyweight and other organs were not affected. The enlarged nephron segments were identified as SGLT2-negative distal segments of proximal tubules and as collecting ducts by histological quantification of tubular cell area. In both segments protein/DNA ratio, a marker for hypertrophic growth, was increased by 6% and 12% respectively, while tubular nuclei number (hyperplasia) was unchanged by empagliflozin. SGLT2-inhibition in early proximal tubules induces a shift of NaCl resorption along the nephron causing compensatory NaCl and H_2_O reabsorption and presumably cell growth in downstream segments. Consistently, in collecting ducts of empagliflozin-treated mice, mRNA expression of the Na^+^-channel ENaC and the H_2_O-channels Aqp-2/Aqp-3 were increased. In addition, the hypoxia marker Hif1α was found increased in intercalated cells of the collecting duct together with evidence for increased proton secretion, as indicated by upregulation of carbonic anhydrases and acidified urine pH in empagliflozin-treated animals. In summary, these data show that SGLT2i induce cell enlargement by hypertrophic growth and possibly cell swelling in healthy kidneys, probably as a result of compensatory glucose, NaCl and H_2_O hyperreabsorption of SGLT2-negative segments. Particularly affected are the SGLT2-negative proximal tubules (S3) and the collecting duct, areas of low O_2_ availability.

## Introduction

SGLT2 inhibitors have substantial beneficial effects on renal and cardiac function in patients with heart failure (McMurray et al., 2019; Packer et al., 2020; Anker et al., 2021) and patients with a wide spectrum of different chronic kidney diseases (CKD) (Heerspink et al., 2020; Herrington et al., 2022). At least since the data of the DAPA-CKD and the recently published EMPA-KIDNEY trial became available, demonstrating impressive renal benefits of SGLT2 inhibition in non-diabetic CKD patients (Heerspink et al., 2020; Herrington et al., 2022), it is obvious that SGLT2 inhibitors by far exceed their initial therapeutic approach of an anti-hyperglycemic agent. Dapagliflozin reduced the risk of sustained GFR decline, end-stage kidney disease or renal death by 44% (Heerspink et al., 2020), for empagliflozin (EMPA) the risk for kidney disease progression declined by 29% (Herrington et al., 2022). However, a clear picture of the underlying mechanisms of renal protection by SGLT2 inhibitors in patients without type II diabetes is still missing.

Several theories have been proposed including blood pressure reduction due to osmotic diuresis and hypovolemia (Kario et al., 2021); a “fasting-like” metabolic shift to increased fatty acid utilization and ketogenesis in response to massive glucose loss (Gao et al., 2022); the protection from glomerular hyperfiltration by a restoration of the tubuloglomerular feedback mechanism (Kidokoro et al., 2019; Thomson and Vallon, 2021) and the protection of proximal tubules from hypoxic injury caused by increased reabsorption load during renal hyperfiltration (Layton et al., 2016). Other beneficial effects of SGLT2 inhibitors, such as the induction of uric acid loss (Novikov et al., 2019), the inhibition of the sympathetic tone (Gueguen et al., 2020; Herat et al., 2020) and a reduction of pro-inflammatory cytokines (Pirklbauer et al., 2020), might contribute to renal protection. However, it should be mentioned that the majority of these findings refer to data from diabetic patients and rodent models with diabetic background. Whether these factors are also relevant in non-diabetic renal protection by SGLT2 inhibitors remains to be elucidated.

Among the proposed hypotheses, long-term stabilization of GFR by protection from hyperfiltration after SGLT2 inhibitor administration appears to be a common nephroprotective mechanism across different clinical trials. The initial event, an immediate dip of the GFR caused by a reduction of intraglomerular pressure in response to the restoration of the tubuloglomerular feedback mechanism (Vallon and Thomson, 2020), was reported for the SGLT2 inhibitor dapagliflozin (Heerspink et al., 2021; Jhund et al., 2021), canagliflozin (Perkovic et al., 2019) and empagliflozin (Wanner et al., 2016; Wanner et al., 2018) in cardiovascular patients with type II diabetes. While early clinical trials did not include non-diabetic patients (Zinman et al., 2015; Neal et al., 2017; Wiviott et al., 2019), recent studies in cardiovascular (Packer et al., 2020) and CKD patients (Heerspink et al., 2021; Herrington et al., 2022) describe a similar reduction of hyperfiltration by SGLT2 inhibitors in patients with non-diabetic nephropathy. The same GFR reducing effect of SGLT2 inhibitors can be seen in hyperfiltrating kidneys of healthy wildtype mice, as we have shown previously (Tauber et al., 2021). Induced by unilateral nephrectomy (UNx), wildtype mice developed compensatory hyperfiltration and EMPA significantly reduced the increased GFR. Remarkably, in kidney disease mouse models in which EMPA did not induce an initial dip of the GFR, no nephroprotection could be observed by EMPA treatment highlighting the importance of GFR control for EMPA-mediated nephroprotection (Tauber et al., 2021).

This “hyperfiltration hypothesis” is closely related to the so-called “workload” hypothesis, which predicts the protection of proximal tubules from reabsorption overload by inhibition of the SGLT2-mediated NaCl and glucose uptake. 99% percent of the filtered glucose and approximately 70% of NaCl present in the primary urine are reabsorbed by the proximal tubule. Cellular reabsorbed glucose and NaCl are exported at the basolateral membrane by the GLUT2 glucose transporter or the Na^+^/K^+^-ATPase respectively. Under conditions of glomerular hyperfiltration, of diabetic or non-diabetic etiology, the resorption capacity of the proximal tubule can be pushed to its maximum. SGLT2 inhibition reduces this hyperstimulation of the early proximal tubule shifting the NaCl and glucose reabsorption workload to further distal tubular segments of the nephron. Chronic increase in GFR and the resulting hyperreabsorption in proximal epithelial cells go hand in hand with compensatory hypertrophic growth of proximal tubules and total kidney mass, as seen in diabetic humans (Mogensen and Andersen, 1973; Wirta and Pasternack, 1995) and rodent models (Seyer-Hansen, 1976; Sharma et al., 1996). Likewise, after UNx in mice, the GFR of the remaining kidney increased by 65% and kidney weight was 20% higher compared to sham-operated control mice only 2 weeks after UNx (Tauber et al., 2021). To our surprise, renal weight gain after UNx was even more pronounced in EMPA-treated animals (+30%) although hyperfiltration was significantly reduced in these mice (83% of UNx mice without EMPA). Additionally, already normofiltrating control mice showed a trend towards kidney weight gain after EMPA treatment. This unexpected observation of functional uncoupling of hyperfiltration and renal growth suggests an independent growth-promoting effect of SGLT2 inhibitors in the kidney. In line with our data, increased kidney weight was observed in SGLT2 knockout animals (Vallon et al., 2013) and other kidney disease rodent models treated with SGLT2 inhibitors (Kapoor et al., 2015; Rajasekeran et al., 2018; Castoldi et al., 2020; Yamato et al., 2020; Castoldi et al., 2021). A possible explanation provides the suggested shift of NaCl and glucose reabsorption to further distal nephron segments in response SGLT2 inhibition in early proximal tubule cells. Stimulated glucose reabsorption in the proximal S3 segment and augmented NaCl uptake in the thick ascending limb, distal tubule and collecting duct possibly triggers compensatory cell growth in these segments. Likewise, interstitial edema formation and/or compensatory H2O reabsorption in response to osmotic diuresis could explain increased kidney weight under SGLT2 inhibition. To address these points, healthy wildtype animals were treated with the SGLT2 inhibitor empagliflozin for 8 weeks and organ weight and hypertrophic cell growth were analyzed using microscopic (quantification of tubular cell size) and molecular approaches (protein/DNA ratio in isolated tubules).

## Material and methods

### Animals and empagliflozin treatment

Adult male C57BL/6 mice (age 11–18 weeks) from Janvier Labs (Le Genest-Saint-Isle, France) were treated for 8 weeks with empagliflozin (EMPA; Carbosynth Limited, Compton, United Kingdom) administered *via* the drinking water at a dose of 30 mg/kg/day. As shown previously, the selected dose results in an EMPA plasma concentration of 46 nM, a concentration within the ideal therapeutic range of EMPA (Tauber et al., 2021). The EMPA concentration was adjusted to the drinking behavior of the animals (8–10 mL/day) and drinking water was replaced every 3–4 days. Animals had free access to water and food. Urine glucose levels (Supplementary Figure S1) were monitored with glucose test strips (Contour XT, Bayer AG, Leverkusen, Germany) to proof the efficiency of EMPA treatment. All experimental procedures were conducted in accordance with the German Animal Welfare Act and approved by the local authorities (government of Lower Franconia, Germany, file number RUF 55.2.2–2532.2-896–13). After 8 weeks EMPA-treated and control animals were killed by cervical dislocation. For immunohistological stainings, one kidney was perfused *via* the abdominal aorta with 0.9% NaCl solution followed by a 4% paraformaldehyde perfusion fixation (3 min; 100 mmHg constant pressure) and paraffin-embedding. Unfixed organs (kidney, heart, brain, lung) were weighted, snap-frozen in liquid nitrogen and stored at −80 °C for RNA extraction.

### Immunofluorescence staining and measurement of tubular area

Paraffin-embedded kidneys were sectioned (5 µm), deparaffinized and stained with primary antibodies for well-established, tubulus-specific marker proteins using a standard paraffin staining protocol. Megalin (sc-515772, Santa Cruz Biotechnology, Dallas, TX, United States) was used as a marker for the proximal tubule S1-S3 segment, Sglt2 (ab85626, abcam, Cambridge, United Kingdom) for the proximal tubule S1/S2 segment, Tamm-Horsfall-protein (THP; sc-271022, Santa Cruz Biotechnology, Dallas, TX, United States) for the loop of Henle, calbindin (Calbindin D-28k; Swant AG, Burgdorf, Switzerland) for the distal convoluted tubule, aquaporin 2 (sc-515770, Santa Cruz Biotechnology, Dallas, TX, United States) for the principle cells of the collecting duct and V-ATPase B1/2 (sc-55544, Santa Cruz Biotechnology, Dallas, TX, United States) for intercalated cells of the collecting duct. After staining with a fluorescence-labeled secondary antibody, whole kidney overview images were acquired using the Zeiss Axio Observer 7 (Carl Zeiss AG, Oberkochen, Germany). As a measurement of cell area per tubule segment, the total area of each 50 segment-specific tubules was quantified using the Zeiss ZEN software (Carl Zeiss AG, Oberkochen, Germany) and divided by the number of DAPI-stained cell nuclei.

### Real-time PCR

RNA was extracted from halved kidneys using the TRIsure™ Kit (BioCat GmbH, Heidelberg, Germany) according to the manufacturer’s recommendation. RNA was quantified and purity was analyzed using the NanoDrop™ 3,300 (Thermo Fisher Scientific GmbH, Dreieich, Germany). Reverse transcription of 2 µg total RNA was conducted using the FastGene® Scriptase Basic cDNA Kit (Nippon Genetics, Düren, Germany) with Oligo dT-primers (Promega GmbH, Walldorf, Germany). cDNA amplification was conducted using the QuantiTect® SYBR® Green PCR Kit (Qiagen, Hilden, Germany) with custom primers for each target gene (Supplementary Table S1). Relative expression of target genes was calculated using 2^−ΔCt^ analysis method. The housekeeper Ct used for normalization was obtained from a pool of Ct values of different housekeeping genes, such as ß-actin, Gapdh, Rpl32 and 18s ribosomal RNA. Data are depicted in relation to the expression level in the H_2_O control group (100%).

### Hif1α staining and quantification

For detection of Hif1α in the kidney, 5 µm sections were deparaffinized and boiled in Target Retrieval Solution (S1699, Agilent, Waldbronn, Germany) for 15 min using a steam pressure pot. After several washing steps sections were blocked with Avidin (SP-2001, Vector, Burlingame, CA, United States) for 20 min, followed by 3% H_2_O_2_ treatment for 10 min and a serum-free protein blocking step (X0,909, Agilent, Waldbronn, Germany) for 60 min. Sections were incubated with the primary antibody against Hif1α (10006421, 1:10,000, Cayman Chemical, Ann Arbor, MI, United States) over night at 4 °C. After incubation with the secondary, HRP-coupled antibody (CS7074, 1:500, Cell Signaling, Danvers, MA, United States) for 45 min, a TSA Plus Biotin reagent (NEL749A001KT, Akoya Biosciences, Marlborough, MA, United States) was added for signal amplification (15 min). Finally, sections were incubated with Streptavidin-HRP conjugate for 30 min (ab64269, abcam, Cambridge, United Kingdom). For the visualization of Hif1α, the HRP substrate DAB (SK-4100, Vector, Burlingame, CA, United States) was added and the formation of the brown precipitate was closely monitored under the microscope until sufficient intensity was obtained and the reaction was stopped by immediate rinsing. For Hif1 α costainings with tubular marker proteins, the additional primary antibodies for megalin, THP, calbindin, Aqp-2 and V-ATPase B1/2 (see section immunofluorescence staining) were visualized by incubation with appropriate Cy5-coupled secondary antibodies for 60 min. For unbiased quantification of Hif1α signal (brown staining) we used the Zeiss ZEN Intellesis Image segmentation software (Carl Zeiss AG, Oberkochen, Germany). In brief, the software uses a machine-learning algorithm for automated identification of stained areas based on an individual training for each tissue class. We defined three different classes recognizing background areas, kidney tissue areas or Hif1α-positive areas.

### Urine pH measurement

Spot urine was collected before the experiment and at week 1, 3, five and seven post EMPA treatment for pH measurement using a standard pH meter (model 766, Knick, Berlin, Germany).

### Kidney microdissection for isolation of single tubules

A second cohort of male C57BL/6 wildtype mice was treated with EMPA (Carbosynth Limited, Compton, United Kingdom) for 2 weeks and kidneys were microdissected for the selection of isolated tubules of the proximal S3 segment and the collecting duct. Briefly, the kidneys were perfused *via* the abdominal aorta with 10 mL incubation solution (140 mmol/L NaCl, 0.4 mmol/L KH_2_PO_4_, 1.6 mmol/L K_2_HPO_4_, 1 mmol/L MgSO_4_, 10 mmol/L sodium acetate, 1 mmol/L α-ketoglutaric acid, 1.3 mmol/L calcium gluconate, 37.5 mg/100 mL glycine, 48 mg/100 mL trypsin inhibitor, pH 7.4 at 37°C) containing 0.1 mg/mL collagenase II (Sigma Aldrich, Taufkirchen, Germany). Sorting solution was prepared by adding 1 mg/mL PVA 4–88 (Sigma Aldrich, Taufkirchen, Germany) to the incubation solution. After perfusion, one kidney was removed, decapsulated, cut into slices and transferred into 1 mL prewarmed collagenase-containing (1 mg/mL) incubation solution in a 37°C thermoshaker (Eppendorf, Wesseling-Berzdorf, Germany) for 10 min at 850 rpm. After adding 1 mL prewarmed incubation solution, 1 mL of the digested tubule solution was transferred into a new tube and kept on ice for sedimentation. This step was repeated 10x every 5 min. Sedimented tubules were washed, resuspended in 2 mL of sorting solution and 100 proximal S3 segments and 100 collecting ducts were separated by visual sorting under a stereomicroscope at 4°C. After collection, samples were centrifuged (5 min, 8,500 rpm, 4°C) and tubule pellets were frozen at −80°C. After thawing, 200 µL autoclaved MilliQ water were added and tubule lysis was initiated by a 60 min incubation in a thermoshaker (37°C), before they were snap-frozen in liquid nitrogen. Samples were thawed again and subjected to ultrasonic treatment (UW 2070; Bandelin electronic, Berlin, Germany) to enhance cell disruption. This freeze/thaw/ultrasound treatment was repeated three times and the supernatant was used for protein and DNA measurement.

### Cell culture

Three immortalized renal cell lines were used in this study as *in-vitro* models for proximal tubule cells (LLC-PK1 cells; provided by Prof. Richard Warth, University of Regensburg, Germany), distal tubule cells (MDCK-C7, provided by Prof. Rainer Schreiber, University of Regensburg, Germany) and collecting duct cells (M-1, provided by Prof. Rainer Schreiber, University of Regensburg, Germany). Porcine LLC-PK1 cells were cultured in DMEM/F12 medium (Thermo Fisher Scientific GmbH, Dreieich, Germany) supplemented with 10% FBS (PAN-Biotech GmbH, Aidenbach, Germany) and 1% penicillin/streptomycin (Capricorn Scientific GmbH, Ebsdorfergrund, Germany). Canine MDCK-C7 cells were cultured in DMEM low glucose medium (Capricorn Scientific GmbH, Ebsdorfergrund, Germany) supplemented with 10% FBS, 1% L-glutamine (Capricorn Scientific GmbH, Ebsdorfergrund, Germany) and 1% penicillin/streptomycin. Murine M-1 cells were cultured in DMEM/F-12/Glutamax medium (Thermo Fisher Scientific GmbH, Dreieich, Germany) supplemented with 10% FBS, 1% penicillin/streptomycin, 1% ITS and 100 nM dexamethasone (Thermo Fisher Scientific GmbH, Dreieich, Germany). The cells were maintained at 37°C under a humid atmosphere of 95% air and 5% CO_2_. Two days before the experiment 1 × 10^5^ (MDCK-C7, LLC-PK1) or 0,75 × 10^5^ (M-1) cells were seeded in 24 well plates and standard medium was replaced by serum-starved medium after 24 h. Next day, cells were stimulated with EMPA 1 µM or TGF-ß 500 p.m. (7754-BH-005, R&D Systems, Minneapolis, MN, United States) in serum-starved medium for 24 h. For cell lysis 400 µL autoclaved MilliQ were added to each well and the plate was incubated at 37°C for 1 h before it was frozen at −80°C. After thawing, cell lysates were transferred to a sterile tube and stored at −20°C until measurement of the protein/DNA ratio was performed.

### DNA and protein quantification

Fluorometric quantification of double-stranded DNA by intercalation of Hoechst 33258 was conducted using the Fluo Reporter® Blue Fluorometric dsDNA Quantitation Kit (Thermo Fisher Scientific GmbH, Dreieich, Germany) following the manufacturer’s guidelines. Calf thymus DNA solution (Sigma Aldrich, Taufkirchen, Germany) with a defined concentration was used to generate a standard curve. Fluorescence intensity of DNA-bound Hoechst 33258 was measured on a Cary Eclipse Fluorescence Spectrophotometer (Agilent, Waldbronn, Germany) using a 360nm/460 nm excitation/emission filter set. Standard colorimetric kits were used for protein quantification of lysates from isolated tubules (740967.50, Macherey-Nagel, Düren, Germany) or cultured cell lines (23235, Thermo Fisher Scientific GmbH, Dreieich, Germany). To compare protein/DNA ratios between different cell lines, data are depicted in relation to the DMSO control group (100%).

### Statistics

Data are shown as mean ± SEM. For single-group comparisons, an unpaired Student’s t-test was used to calculate the level of significance. Accordingly, for multi-group comparisons at different time points, a two-way ANOVA followed by a Bonferroni *post hoc* test was used. All statistical analyses were performed using the GraphPad Prism software. Differences between groups were considered significant at a *p* < .05.

## Results

In a previously published study by our group, wildtype mice with unilateral nephrectomy (UNx)/DOCA/salt-induced renal injury showed an increase in renal weight under SGLT2 inhibition by empagliflozin (EMPA) (Tauber et al., 2021). Renal weight gain was also observed in healthy control mice and was therefore not related to the compensatory hyperfiltration elicited by UNx. Here, we used healthy wildtype animals to investigate how EMPA affects kidney weight in normofiltrating kidneys. Histological quantification of tubular cell area was performed to identify enlarged nephron segments and protein/DNA ratio was measured in isolated tubules as a marker for cellular hypertrophy.

### Empagliflozin increases kidney weight

**FIGURE 1:**
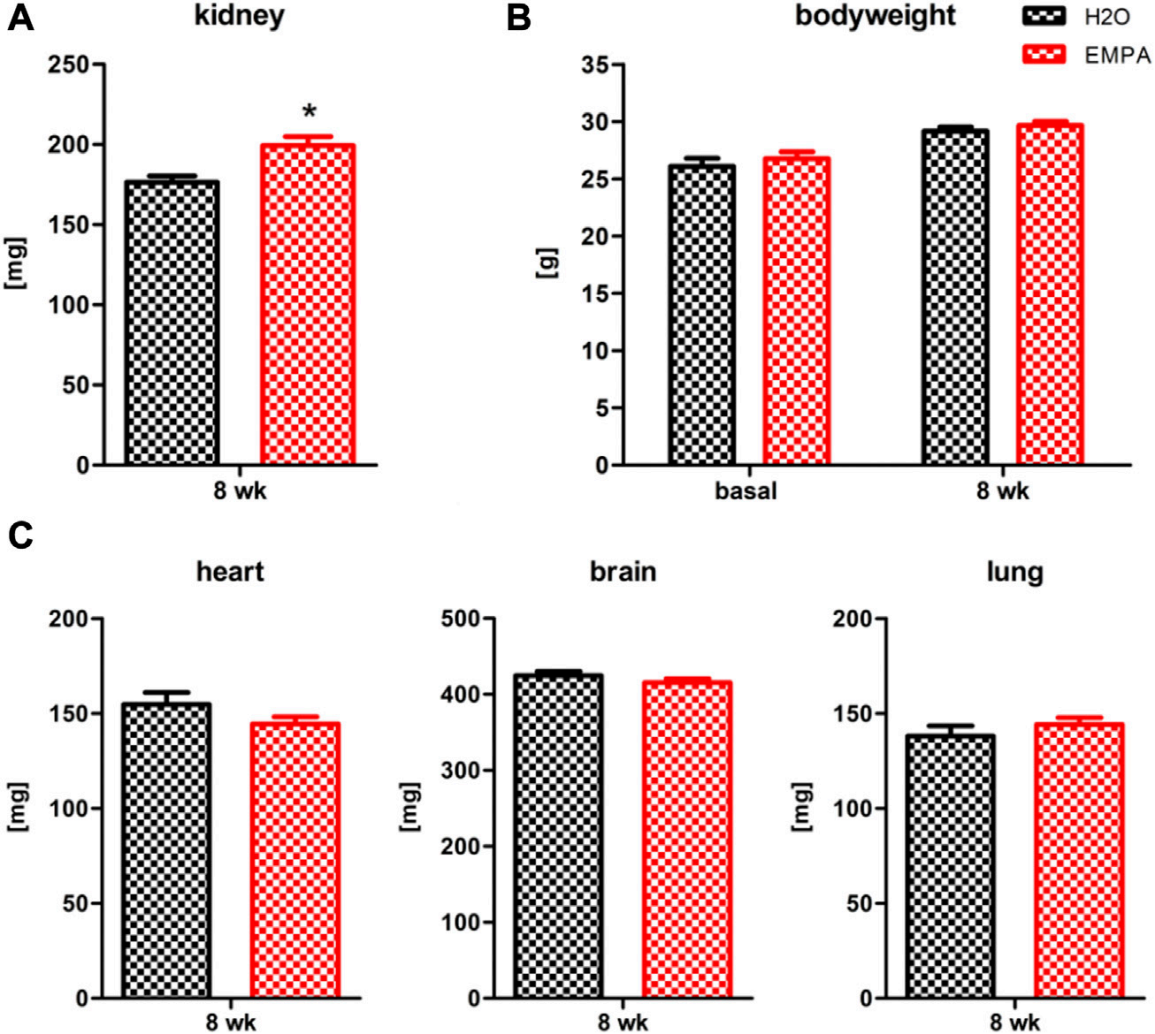
Increased kidney weight in EMPA-treated mice. Wildtype animals were treated with EMPA (30 mg/kg/d; *n* = 16) for 8 weeks and untreated animals were used as control (*n* = 12). Kidney weight **(A)** was increased in EMPA-treated animals compared with H_2_O control animals, while there were no differences in bodyweight **(B)**, heart, brain or lung weight **(C)**. Bar charts show mean values (±SEM) and asterisks indicate *p* < 0.05. EMPA, empagliflozin; wk, weeks.

Treatment of wildtype mice with EMPA for 8 weeks caused massive glucosuria, reduced urine creatinine levels (Supplementary Figure S1) and a significant 13% increase (Figure 1A) in kidney weight (176,43 ± 3.95 mg) compared with H_2_O control animals (199,27 ± 5.72 mg). However, EMPA did not affect the GFR of the animals (Supplementary Figure S6). Body weight increased slightly in both experimental groups within the 8 week period, but no difference was observed between H_2_O and EMPA treatment after 8 weeks (Figure 1B). In other organs, such as heart, brain and lung (Figure 1C), SGLT2 inhibition did not affect organ weight, excluding a general effect of EMPA on organ weight.

### Enlargement of Sglt2 negative proximal tubules (S3 segment) and collecting ducts in EMPA-treated kidneys

**FIGURE 2:**
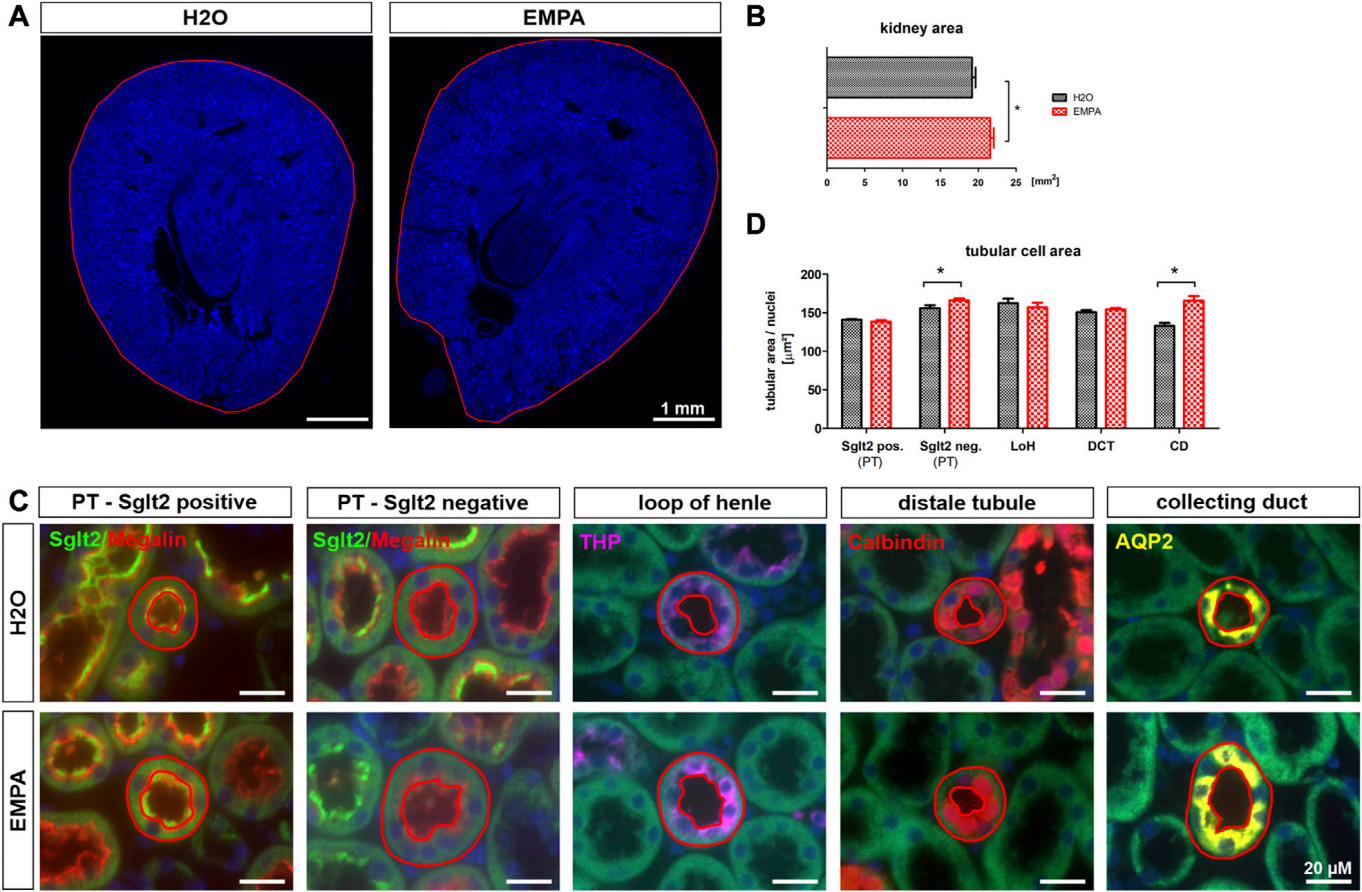
Increased tubular size in EMPA-treated mice. **(A)** Kidney overview images (DAPI staining) demonstrate increased kidney size **(B)** in 8-week EMPA-treated animals (30 mg/kg/d). **(C)** Representative immunofluorescence staining of different nephron segments of EMPA treated animals (*n* = 8; H_2_O animals *n* = 4). Proximal tubule EMPA-target cells (S1/S2) show positive megalin (red) and Sglt2 (green) co-staining. Megalin positive, Sglt2 negative tubules represent the proximal tubule S3 segment. Tamm-Horsfall protein (THP; purple) was used as marker for the thick ascending limb, calbindin (orange) as marker for the distal tubule and aquaporin-2 (Aqp-2; yellow) as marker for the collecting duct. DAPI (blue) was used for cell nuclei staining. Red circles show exemplarily which tubules were included in the analysis shown in **(D) (D)** Quantification of tubular cell area per nephron segment. Only tubules with vertical orientation were considered for cell area quantification (red circles). Tubule area, excluding the luminal space, was normalized to the number of DAPI positive cell nuclei per tubule. Megalin positive tubules were further subclassified in Sglt2 positive and negative proximal tubules. Bar charts show mean values (±SEM) and asterisks indicate *p* < 0.05. EMPA, empagliflozin; PT, proximal tubule; LoH, loop of Henle; DCT, distal convoluted tubule; CD, collecting duct.

Increased organ weight can be a result of water retention in the interstitial space (edema) or an increase in cell mass due to stimulated cell division (hyperplasia) or cell growth (hypertrophy). The kidney wet-to-dry weight ratio was not significantly affected by EMPA treatment (Supplementary Figure S2), which is why edema formation seems unlikely to be causative for increased kidney weight. On a macroscopic level, EMPA increased kidney size (Figure 2A) as quantified by measuring whole kidney area (Figure 2B).In order to investigate cellular effects, kidneys were stained for marker proteins of different nephron segments (Figure 2C) and total tubular area and number of nuclei per stained tubule were quantified for each nephron segment. The ratio of tubular area/number of nuclei was used as a measure of the actual cell size. To further subclassify the proximal tubule into EMPA target and non-target cells, Sglt2 positive/megalin positive tubules were defined as S1/S2 segments (EMPA-target), and Sglt2 negative/megalin positive tubules as S3 segments (EMPA non-target). Kidneys of EMPA-treated animals showed a significantly enlarged cell area in Sglt2 negative/megalin positive proximal tubules (6% increase) and Aqp-2 positive collecting ducts (25% increase) compared to control animals (Figure 2D). No difference in cell size was observed in Sglt2 positive/megalin positive tubules, tubules of the thick ascending limb (THP positive) and the distal convoluted tubule (calbindin positive). The number of cell nuclei per tubule did not differ in any nephron segment between H_2_O and EMPA treated animals (Supplementary Figure S3), which is why the hyperplasia hypothesis was not further pursued. Hyperplastic growth is defined by an increase in cell number due to stimulated proliferation and DNA amplification resulting in a higher number of cell nuclei per tubule.

### Hypertrophic cell growth in S3 segments and collecting ducts of EMPA-treated mice

**FIGURE 3:**
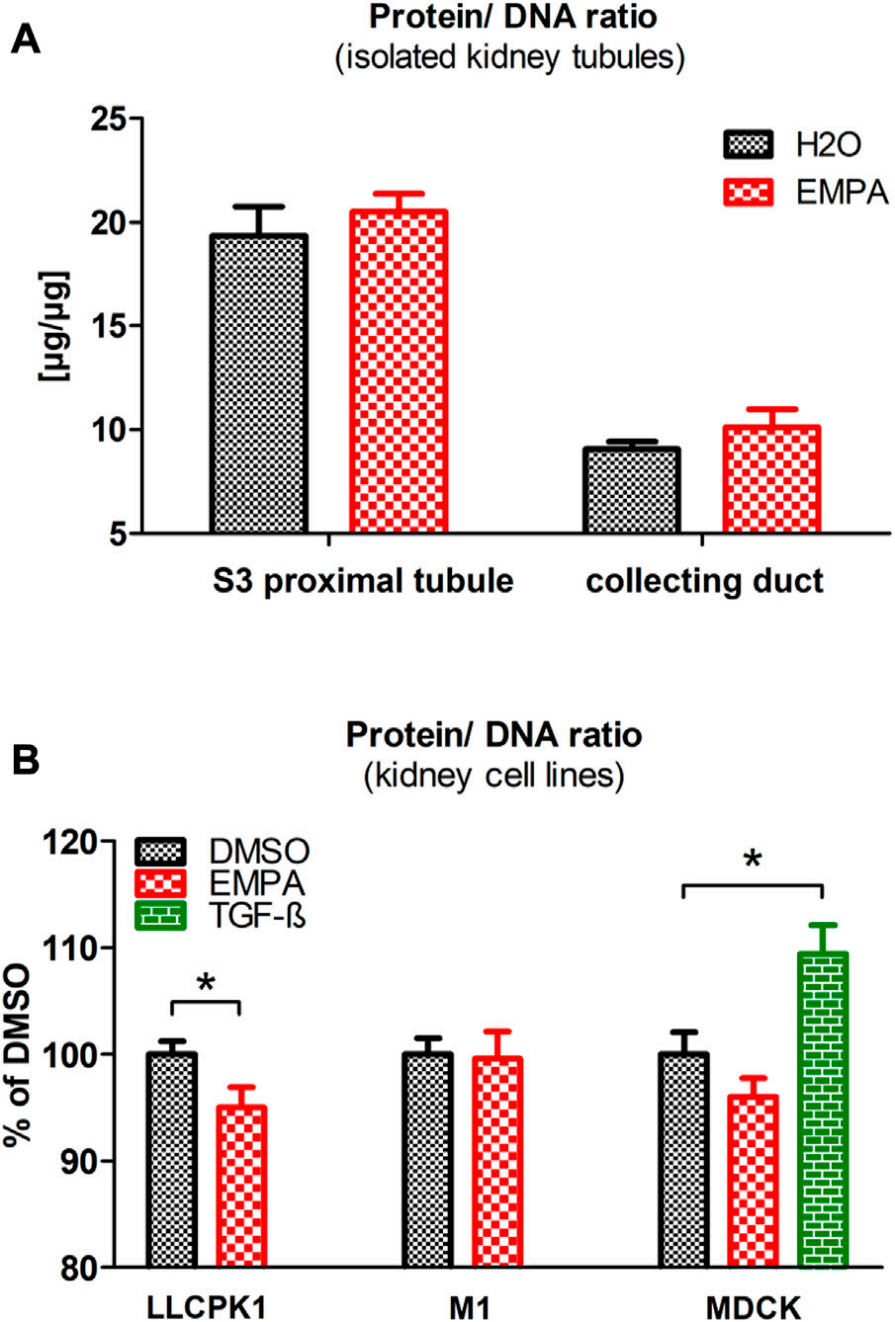
Protein/DNA ratio in isolated tubules and renal cell lines after EMPA treatment. **(A)** Microdissected proximal S3 segments and collecting ducts of EMPA-treated mice (30 mg/kg/d for 8 weeks; *n* = 11) showed a slight, non-significant increase of the protein/DNA ratio compared with H_2_O control mice (*n* = 10). **(B)**. Impact of EMPA on protein/DNA ratio in three renal cell lines (*n* = 7–11 with three to six replicates each). After 24 h EMPA treatment (1 µM), protein/DNA ratio was significantly reduced in LLC-PK1 cells. In MDCK cells the EMPA-induced reduction of the protein/DNA ratio was not statistically different to control cells. As an activator of cell cycle arrest, TGF-ß (500 p.m.), used here as positive control, increased the protein/DNA ratio in MDCK cells. To compare protein/DNA ratios between different renal cell lines, data are depicted in relation to the expression level in the DMSO control group (100%). Bar charts show mean values (±SEM) and asterisks indicate *p* < 0.05. EMPA, empagliflozin; DMSO, Dimethylsulfoxid; TGF-ß, Transforming growth factor beta.

An increase in cell volume can be due to two causes, hypertrophic cell growth or fluid-based cell swelling. To further explore these two options, we measured the protein/DNA ratio, as a marker for cellular hypertrophy, in proximal tubule S3 segments and collecting ducts of EMPA-treated mice, isolated by microdissection. In case of hypertrophic cell growth the intracellular amount of protein increases whereas the amount of DNA remains constant, while in cell swelling both parameters would be diluted resulting in unaltered protein/DNA ratio. Microdissection was performed on kidneys of untreated wildtype mice and mice treated with EMPA for 2 weeks, since we have shown in a previous study that renal weight gain occurs already after a 2 weeks EMPA treatment (Tauber et al., 2021). In both the EMPA group and the control group without EMPA, the protein/DNA ratio in isolated proximal tubule S3 segments was almost two-fold higher compared to the protein/DNA ratio in the collecting ducts (Figure 3A). EMPA treatment raised the protein/DNA ratio by 6% in isolated S3 segments and by 12% in collecting ducts. However due to high variation within the groups, the difference to untreated control animals did not reach the level of significance. To evaluate whether the impact of EMPA on hypertrophic growth relies on direct, SGLT2-mediated actions or indirect, off-target effects, EMPA was applied to three renal cell lines (LLC-PK1, MDCK and M-1) and protein/DNA ratio was measured. As a model for EMPA target cells, LLC-PK1 cells, the only cell line used in this study with relevant expression levels of SGLT2 (Supplementary Figure S4), showed a significant lower protein/DNA ratio after EMPA treatment (Figure 3B). No significant differences after EMPA treatment were observed for cell lines lacking SGLT2 expression (Figure 3B), M-1 (model for collecting duct cells) and MDCK cells (model for the loop of Henle). As a positive control, TGF-ß significantly increased the protein/DNA ratio in MDCK cells, which showed the highest expression for TGF-ß receptors among the 3 cell lines (Supplementary Figure S4).

### Increased expression of transporter proteins in collecting ducts of EMPA-treated mice

**FIGURE 4:**
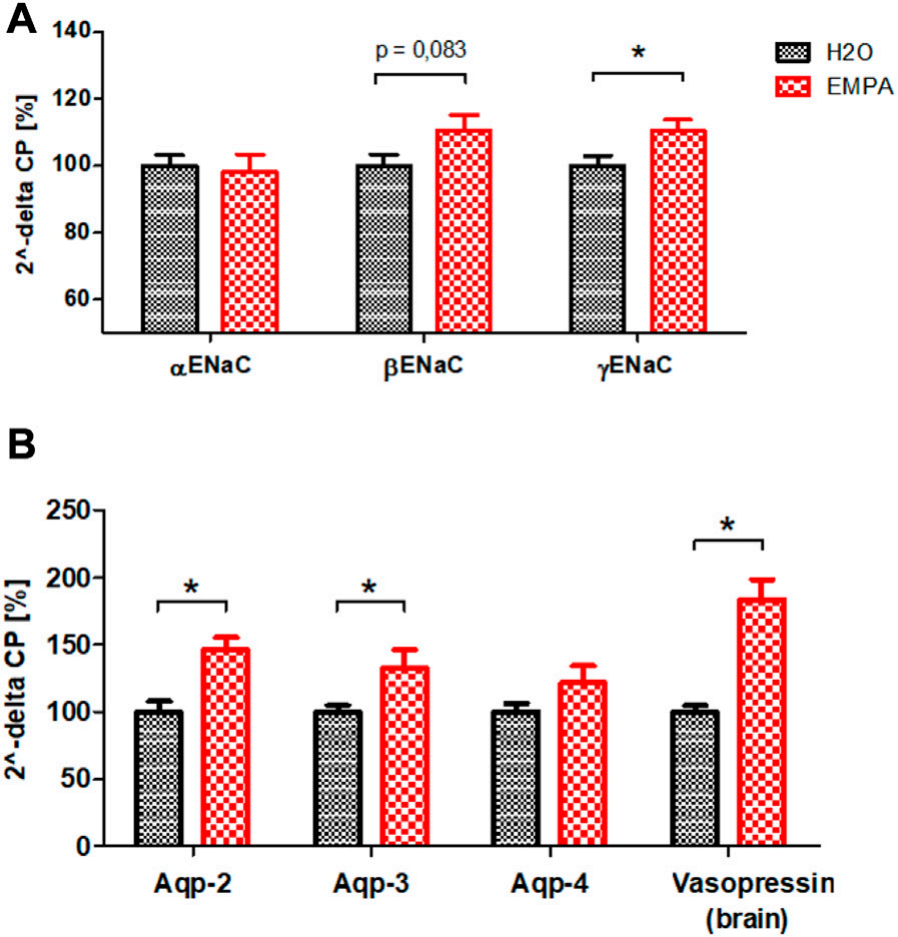
mRNA quantification of sodium channel ENaC subunits **(A)** and aquaporins in kidneys and vasopressin in brains **(B)** of untreated wildtype mice (*n* = 8) and mice treated with EMPA (30 mg/kg/d; *n* = 8) for 8 weeks. Bar charts show mean values (±SEM) and asterisks indicate *p* < 0.05. EMPA, empagliflozin; Aqp-2, aquaporin-2; Aqp-3, aquaporin-3; Aqp-4, aquaporin-4.

EMPA reduces glucose and sodium uptake in the S1/S2 segment of the proximal tubule. Most likely, as a compensatory mechanism, tubular segments downstream of the S1/S2 segment increase their NaCl resorption capacity, while glucose reabsorption is limited to SGLT1-driven uptake in the S3 segment of proximal tubules. Because cell size increment under EMPA treatment was most pronounced in cells of the collecting duct (Figure 2), mRNA transcription profiles of the Na^+^ channel ENaC and H_2_O permeable aquaporins were analyzed. Among the three subunits of the ENaC channel, the mRNA abundance of the ß- and γ-subunit was enhanced (11% and 10% increase) in EMPA-treated animals compared to control animals, but only the expression difference of the γ-subunit reached the significance threshold (Figure 4A). However, this increase was not found on protein level (Supplementary Figure S5). mRNA levels of the H_2_O channels aquaporin-2 (Aqp-2; 46% increase), located in the luminal cell membrane, and aquaporin-3 (Aqp-3; 33% increase), located in the basolateral cell membrane, were significantly higher in kidneys of EMPA mice (Figure 4B). A similar, but non-significant trend was observed for the basolateral located H_2_O channel aquaporin-4 (Aqp-4; 21% increase). In addition, mRNA level of vasopressin (ADH), main regulator of Aqp-2 and Aqp-3 in the kidney but synthesized in the hypothalamus, was increased in brains of EMPA-treated mice by 84% (Figure 4B).

### Empagliflozin induces Hif1α expression in intercalated collecting duct cells

**FIGURE 5:**
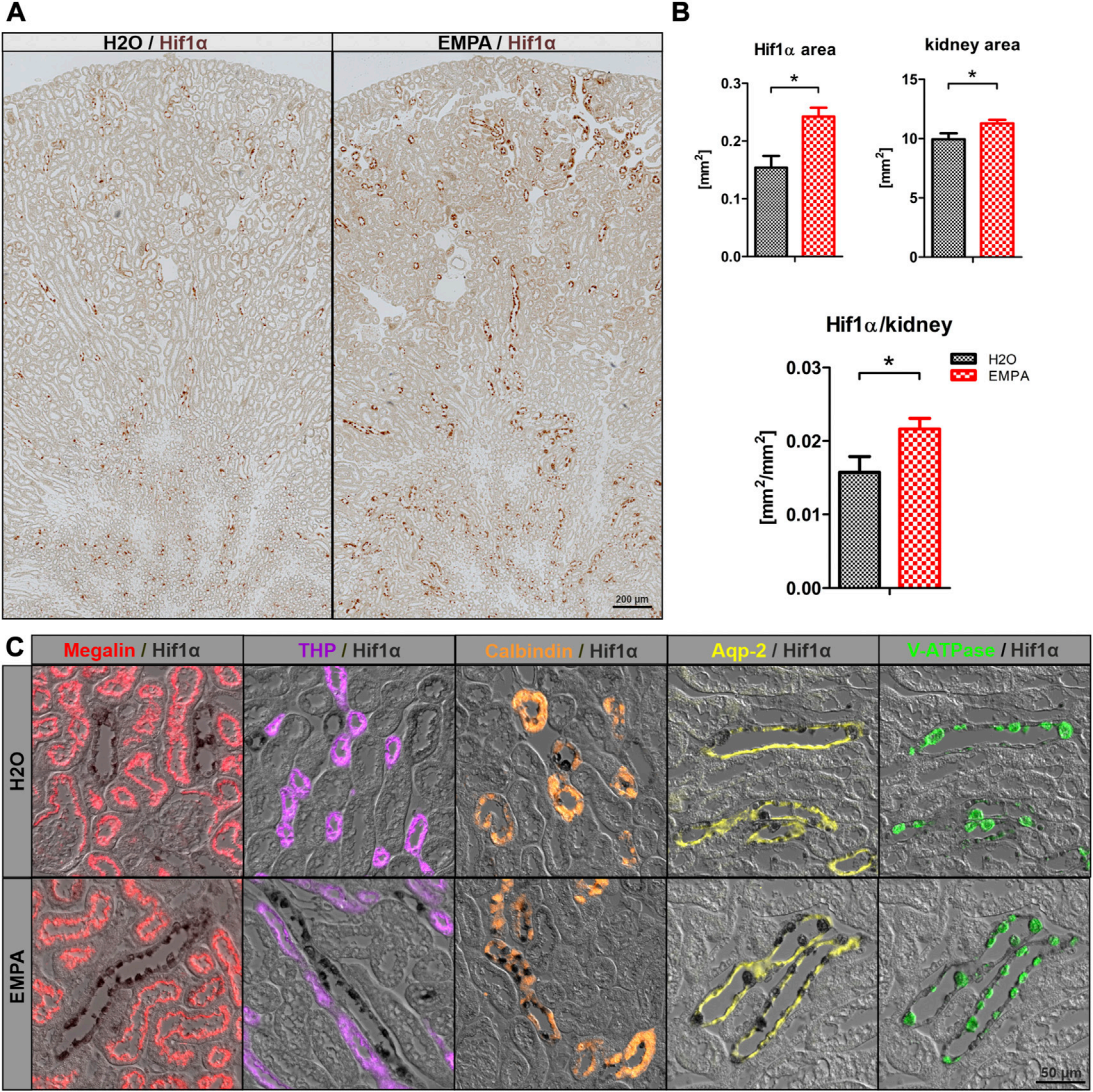
Induction of Hif1α in intercalated cells by SGLT2 inhibition. **(A)** Overview images of Hif1α stained kidneys (brown) of untreated wildtype mice (*n* = 4) and 8 weeks EMPA-treated mice (30 mg/kg/d; *n* = 8). **(B)** Quantification of total kidney Hif1α positive areas, total tubule tissue area and the resulting ratio using the ZEN Intellesis image segmentation software. **(C)** Immunofluores-cence costaining of Hif1α (black) with marker proteins for the proximal tubule (megalin; red), thick ascending limb (Tamm-Horsfall protein, THP, purple), distal convoluted tubule (calbindin; orange), principle (Aqp-2; yellow) and intercalated (V-ATPase; green) cells of the collecting duct. Hif1α was found in some calmodulin positive tubules but mostly in V-ATPase positive intercalated cells of cortical and medullary collecting ducts. Bar charts show mean values (±SEM) and asterisks indicate *p* < 0.05. EMPA, empagliflozin.

Na^+^ reabsorption in the kidney is a highly energy-demanding process, as the export of sodium out of the cell is tightly coupled to the activity of the basolateral located Na^+^/K^+^-ATPase. Increased Na^+^ uptake due to higher ENaC abundance might potentially exacerbate the already poor oxygen supply to the collecting duct cells and provoke an oxygen deficit in these cells. Therefore, Hif1α, an early marker for hypoxic conditions, was stained in kidney sections of EMPA-treated wildtype mice and control animals. Overview images revealed Hif1α -positive cells in renal tubules in both groups, primarily located in the inner cortex and outer medulla (Figure 5A). Visual inspection suggested slightly more Hif1α abundance in cortical tubules of kidneys from EMPA-treated mice compared to control mice. Automated quantification of Hif1α areas on total kidney tissue (Figure 5B) confirmed an increase in Hif1α protein expression under SGLT2 inhibition both in absolute numbers (57% increase) and normalized to total kidney size (37% increase). Hif1α signal was not detected in proximal tubules stained with megalin (red; Figure 5C) and tubules of the thick ascending limb stained with Tamm-Horsfall protein (THP; purple; Figure 5C). Some calmodulin-positive tubules (orange; Figure 5C) showed Hif1α expression, but the majority of Hif1α positive cells were located in Aqp-2 positive collecting ducts (yellow; Figure 5C) in control and EMPA-treated animals. Interestingly, co-staining with V-ATPase (green; Figure 5C) identified most Hif1α positive cells as intercalated cells of the collecting duct. In contrast, Hif2α was ubiquitously expressed in the kidney with no difference between EMPA and control animals (Supplementary Figure S8).

### Urinary acidification in EMPA-treated mice

**FIGURE 6:**
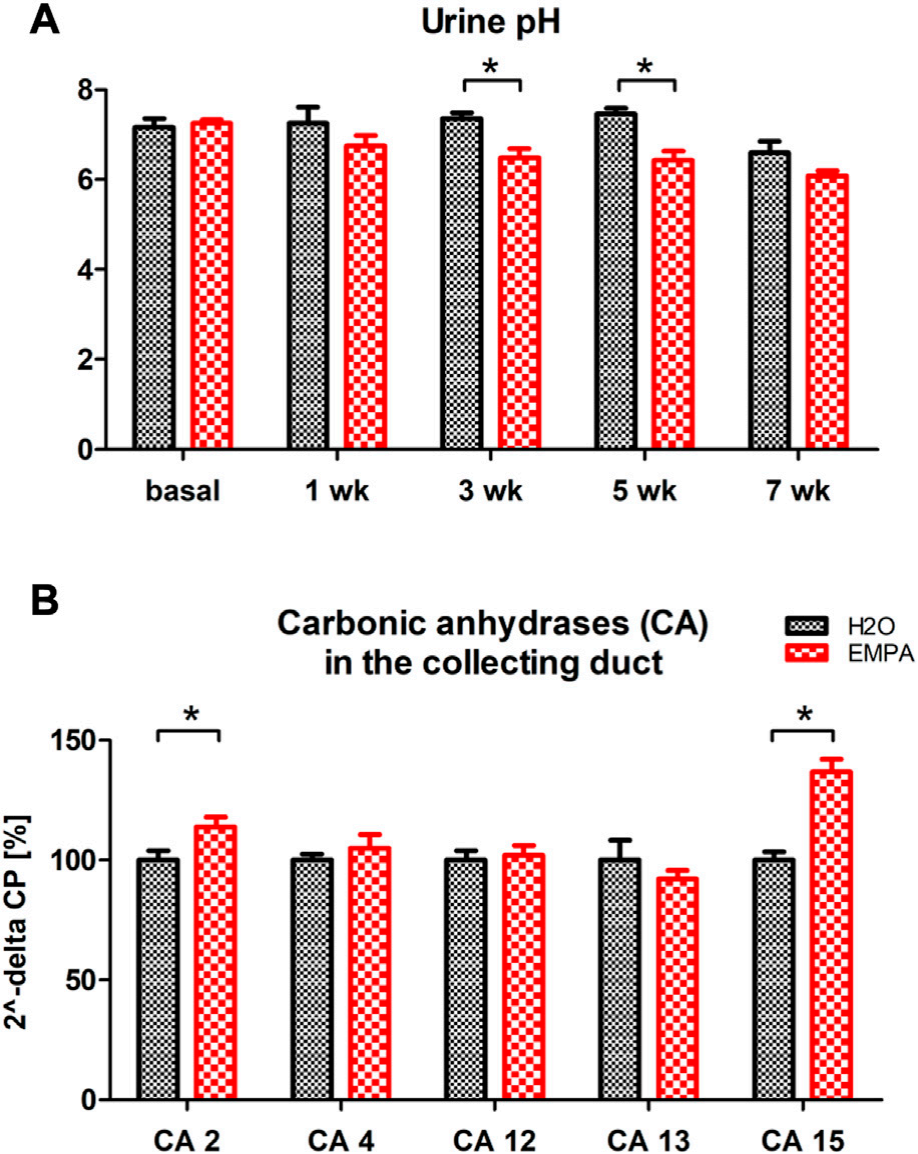
Urine pH and kidney mRNA expression of carbonic anhydrases in EMPA-treated animals. Wildtype animals were treated with EMPA (30 mg/kg/d; *n* = 8 per group) for 8 weeks **(A)** Spot urine pH was more acidified in EMPA-treated animals compared to control mice at all timepoints. **(B)** mRNA quantification of selected carbonic anhydrases in kidneys of EMPA-treated mice. Bar charts show mean values (±SEM) and asterisks indicate *p* < 0.05. EMPA, empagliflozin; wk, weeks; CA, carbonic anhydrase.

Increased ENaC expression in principle cells and Hif1α stabilization in intercalated cells suggest an activation of transport processes in the collecting duct of EMPA-treated mice. Intercalated cells secrete protons and bicarbonate to the urine in response to changes in blood pH preventing systemic acidosis or alkalosis. To test intercalated cell function during EMPA therapy, we measured pH in spot urine from EMPA-treated animals over 8 weeks. Urinary pH was lower in EMPA-treated animals throughout the 8 weeks treatment period. At week 3 and 5 pH was significantly decreased in EMPA-treated animals (Figure 6A). Consequently, mRNA expression level of pH regulating genes of intercalated cells (V-ATPases, H^+^/K^+^-ATPases, carbonic anhydrases, chloride-bicarbonate exchanger) was quantified in EMPA-treated animals (Figure 6; Supplementary Table S2). Among the tested gene families, transcriptional regulation was only observed for two members of the carbonic anhydrases expressed in intercalated cells. Cytosolic carbonic anhydrase 2 (CA2) mRNA was significantly higher (14%) in kidneys of EMPA-treated mice (Figure 6B). In addition, carbonic anhydrase 15 (CA15), a poorly studied member of the membrane-bound carbonic anhydrases, presumably involved in luminal acid-base handling of renal intercalated cells (Saari et al., 2010) was upregulated by 37% in kidneys of EMPA-treated wildtype mice (Figure 6B). Other membrane-bound (CA 4, CA12) or cytosolic (CA13) carbonic anhydrases, known to be expressed in the collecting duct, were not regulated on mRNA level (Figure 6B).

## Discussion

An increase in kidney size can be seen in a number of physiological and pathological situations, including diabetes mellitus (Wolf and Ziyadeh, 1999), unilateral nephrectomy (Nowinski, 2002; Chen K. W. et al., 2016), congenital growth abnormalities of the kidney (McArdle et al., 2020) and obesity (Tobar et al., 2013). Renal growth primarily occurs in glomeruli and cortical tubules (Wolf and Ziyadeh, 1999) as a compensatory response to a loss of healthy nephrons either by nephrectomy or kidney injury and can be seen as the structural adaption to diabetic or non-diabetic glomerular hyperfiltration (Helal et al., 2012). In the present study, an increase in kidney mass was observed in healthy wildtype animals after SGLT2 inhibition by empagliflozin (EMPA) (Figure 1A). Kidney function, as measured by GFR was not affected by EMPA treatment (Supplementary Figure S6) in these animals, which is in line with our previously published GFR data from sham-operated control animals in our UNx/DOCA/salt study (Tauber et al., 2021).

Several studies in different kidney injury models describe a similar increase in kidney weight for empagliflozin (Vallon et al., 2014; Castoldi et al., 2020; Hojná et al., 2022) and dapagliflozin (Kapoor et al., 2015; Rajasekeran et al., 2018) in healthy control mice, but GFR data are often missing and no one has elucidated the underlying mechanisms yet. Hence, the current work focused on the localization and classification of renal growth in response to SGLT2 inhibition. SGLT2 inhibitors are thought to shift NaCl reabsorption load from proximal S1/S2 segment to downstream nephron segments causing compensatory hyperreabsorption, increased expression of salt transporters and possibly cell growth (Vallon and Thomson, 2020). In healthy wildtype animals, EMPA induced an expansion of cell area in proximal tubule S3 segments and, to our surprise, in cells of the collecting duct (Figure 2). Cell enlargement emerges from tubular hypertrophy or fluid-induced cell swelling. In type I and type II diabetes proximal tubule growth is a multistep event with early proliferation of epithelial cells (hyperplasia) followed by subsequent cell cycle arrest and cellular hypertrophy (Uehara-Watanabe et al., 2022). In comparison, compensatory growth of proximal epithelial cells following unilateral nephrectomy is hypertrophic, not hyperplastic, evidenced by increased protein production with unchanged DNA synthesis rate (Liu and Preisig, 2002). The enlarged tubules of the S3 segment and collecting duct in EMPA-treated mice showed no difference in the number of cell nuclei (Supplementary Figure S3) excluding hyperplastic growth in these segments. Protein/DNA ratio was numerically increased in isolated S3 segments and collecting ducts of EMPA-treated mice (Figure 3A), even though the difference did not reach the significance level. These rather small effects on the protein/DNA ratio might be explained by the fact, that S3 and collecting duct tubules were isolated from mice treated with empagliflozin for only 2 weeks. It is likely that hypertrophic growth continues to increase with prolonged EMPA administration and thus differences in protein/DNA ratio become more pronounced after 8 weeks, when histological quantification of tubular area was performed. In addition, cell culture experiments in different renal cell lines argue against an off-target effect of EMPA as reason for hypertrophic growth of collecting duct cells. Actually, the only SGLT2 expressing cell line in our study, LLC-PK1 cells, showed a reduced protein/DNA ratio after EMPA stimulation suggesting a hypotrophic effect of EMPA on SGLT2 target cells (Figure 3B). Hypotrophy may be explained by reduced transport activity as a result of SGLT2 inhibition. A similar hypertrophic effect as found in collecting ducts of EMPA-treated animals, is described for the distal tubule, connecting tubule and collecting duct in furosemide-infused rats (Kaissling and Stanton, 1988; Stanton and Kaissling, 1988). Diuretics-induced hypertrophy is often coupled with enhanced Na^+^ reabsorption downstream from the site of diuretic action as shown by increased abundance of NKCC2, NCC and ENaC as response to chronic infusion of furosemide or hydrochlorothiazide in rats (Na et al., 2003). In correlation with these findings, the mRNA expression of ENaC was increased in enlarged collecting ducts of EMPA-treated animals (Figure 4A) supporting the proposed concept of increased reabsorption workload in this segment. The rather small effect on mRNA expression of the ENaC subunits was not found on protein level (Supplementary figure S5) and the literature is very contradictory regarding the regulation of renal sodium transporters by SGLT2 inhibitors ranging from no effect (Chen L. et al., 2016; Kravtsova et al., 2022), reduced expression (Chung et al., 2019) or increased expression (Ma et al., 2019). It has to be mentioned that only this study used healthy wildtype animals, while most expression analysis data derive from rodent disease models for diabetes or salt-induced hypertension, treatments known to affect renal Na^+^ handling itself.

In addition to increased ENaC expression (Figure 4A), H_2_O-permeable aquaporins Aqp-2 and Aqp-3 were transcriptionally upregulated in EMPA-treated animals (Figure 4B), possibly as a compensatory physiological response to SGLT2 inhibitor induced diuresis to stabilize body fluid volume (Ansary et al., 2017; Masuda et al., 2020). Aqp-2 and Aqp-3 are mainly regulated by vasopressin. Aqp-2 and Aqp-3 are mainly regulated by vasopressin. Vasopressin is synthesized in the hypothalamus and released into the blood from the neurohypophysis. Indeed, vasopressin mRNA was increased in the brains of EMPA-treated mice (Figure 4B). Even though, 24-h urine volume and total body fluid was not assessed in this study, high glucose and low creatinine concentrations in spot urine samples of EMPA-treated mice (Supplementary Figure S1) speak for an osmotic diuresis in these animals. These findings are in line with a recently published study describing an increased workload in the collecting duct of healthy and diabetic rats during SGLT2 inhibition shown by increased vasopressin-induced Aqp-2 expression caused by osmotic diuresis (Masuda et al., 2022). Therefore, fluid-induced cell swelling might contribute to the enlarged cells size of collecting duct cells in EMPA-treated mice, in addition to hypertrophic growth.

In cells of the proximal tubule S3 segments, mRNA expression of the glucose transporter SGLT1 was not significantly altered (Supplementary Figure S5) under SGLT2 inhibition, as shown by other groups before (Gangadharan Komala et al., 2014; Vallon et al., 2014; Chen et al., 2020). However, Rieg et al. demonstrated that glucose transport through SGLT1 is more than 10-fold increased during genetic or pharmacological inhibition of SGLT2 in mice (Rieg et al., 2014), suggesting posttranscriptional regulation of SGLT1 in response to EMPA treatment. SGLT2 inhibitors exonerate the early proximal tubule from glucose-linked Na^+^ reabsorption and possibly also from NHE3-mediated Na^+^ uptake evidenced by increased inhibitory phosphorylation of NHE3 in EMPA-treated mice and rats (Onishi et al., 2020; Borges-Júnior et al., 2021). This shift of ATP-consuming NaCl reabsorption to downstream nephron segments ensures early proximal tubule integrity, but at the same time increases the risk of developing hypoxia in the inner cortex and outer medulla, areas with already low O_2_ availability (Layton et al., 2015; 2016). Indeed, reduced medullary P_O2_ levels were measured in healthy and diabetic anaesthetized rats after non-selective inhibition of SGLT1 and SGLT2 by phlorizin, while cortical P_O2_ was unchanged by phlorizin administration in healthy rat kidneys (O'Neill et al., 2015). For cellular markers of hypoxia, the work published so far describes renal Hif1α suppression by SGLT2 inhibitors in proximal tubules of diabetic mice (Cai et al., 2020) or cultured human renal proximal tubular epithelial cells (Bessho et al., 2019). This study shows, for the first time, increased Hif1α expression in the inner cortex and outer medulla (Figure 5A, B) of EMPA-treated wildtype animals. Hif1α was primarily localized in cells of the cortical collecting duct, and possibly the connecting tubule. Most Hif1α protein was found in intercalated cells of the collecting duct, compared to rather low Hif1α abundance in ENaC-expressing principal cells (Figure 5C). Hif2α, however, the major regulator of erythropoietin transcription was not regulated by EMPA in our animals (Supplementary Figure S8).

α-intercalated cells contribute to urinary acidification by the luminal secretion of protons *via* H^+^- or H^+^/K^+^-ATPases. Postulating that increased Hif1α is an indicator for excessive ATP-consuming proton secretion in these cells, the urine of EMPA-treated animals was indeed more acidified compared to control animals (Figure 6A). Among the pH-regulating genes of α-intercalated cells (V-ATPases, H^+^/K^+^-ATPases, carbonic anhydrases, chloride-bicarbonate exchanger; Supplementary Table S4) only carbonic anhydrases 2 and 15 were transcriptionally upregulated in EMPA-treated kidneys (Figure 6B) which possibly promote urine acidification in EMPA animals by generating protons in intercalated cells. A cidic urinary pH has been described in healthy volunteers after a 4 weeks empagliflozin treatment (Harmacek et al., 2022) and for canagliflozin in patients with type 2 diabetes (Li et al., 2020). The authors suspected an inhibitory effect of canagliflozin on proximal tubule NHE3 activity, for which a possible interaction with SGLT2 inhibitor empagliflozin has been described in NHE3-KO mice (Onishi et al., 2020). In NHE3 wildtype control mice of the same study, acute EMPA treatment for 3 h increased urine pH and bicarbonate excretion, as described for anesthetized rats after acute luseogliflozin injection (Ansary et al., 2017). In contrast, chronic EMPA treatment over 20 weeks reduced urine pH (Onishi et al., 2020), which correlates with our findings. This discrepancy between early and chronic effects on urinary pH might be explained by a slowly progressing systemic acid load as response to the urinary loss of glucose and calories induced by SGLT2 inhibition. The immense glucosuria induced by 30 mg/kg/day EMPA treatment (Supplementary Figure S1) presumably triggers a systemic, fasting-like, metabolic switch with increased lipolysis, lipid oxidation and formation of ketone bodies like 3-hydroxybutyrate, which probably also affects urine pH homeostasis (Ferrannini et al., 2016; Kim et al., 2019; Mulder et al., 2019; Nishimura et al., 2019). In addition, reduced NaCl uptake in the early proximal tubule with subsequent increased NaCl reabsorption in distal segments like the collecting tube possibly affects the acid-base handling of the intercalated cells in response to increased ENaC activity in principle cells. Na^+^ reabsorption by ENaC increases the driving force for K^+^ secretion *via* ROMK into the urine, which can be reabsorbed by the H^+^/K^+^-ATPases in exchange for a proton causing urinary acidification. However, ENaC uptake assays and metabolic profiling was not performed in this study and further experiments are needed to address these issues in wildtype mice under SGLT2 inhibition. In summary, this study addressed the mechanism underlying renal weight gain in the healthy kidney during SGLT2 inhibitor treatment. Hypertrophic growth was observed in the proximal S3 segment and the collecting duct. In the collecting tubule, fluid induced cell-swelling seems likely as an additional cause for the microscopically quantified increase in tubular cell volume. For the first time, an induction of Hif1α by empagliflozin was described in intercalated cells of healthy kidneys possibly as a consequence of increased ATP-demanding proton secretion.

## Data Availability

The raw data supporting the conclusion of this article will be made available by the authors, without undue reservation.

## Abbreviations

CKD, Chronic kidney disease; EMPA, Empagliflozin; FITC, Fluorescein-isothiocyanate; GFR, Glomerular filtration rate; UNx, Unilateral nephrectomy.
