# Supplementary material for: Empagliflozin increases kidney weight due to increased cell size in the proximal tubule S3 segment and the collecting duct
Source: Front Pharmacol. 2023 Mar 23;14:1118358. doi: 10.3389/fphar.2023.1118358 (PMC10076569; doi:10.3389/fphar.2023.1118358)
Supplement: Supplementary file 1 [file DataSheet1.docx]

Supplementary Material

Empagliflozin increases kidney weight due to increased cell size in the proximal tubule S3 segment and the collecting duct

**Frederick Sinha, Anna Federlein, Annika Biesold, Magdalena Schwarzfischer, Katharina Krieger, Frank Schweda*, Philipp Tauber***

* Correspondence: Philipp.Tauber@ur.de; Frank.Schweda@ukr.de

# Supplementary Tables

**Supplemental table 1. Custom primers used with SYBR Green qPCR**

| Gene | Species | Forward (5´-3´) | Reverse (5´-3´) |
| --- | --- | --- | --- |
| αENaC | mouse | tcctgcttccaggagaacat | cctgcagtttatagtagcagtagcc |
| ßENaC | mouse | ttcaactggggcatgacag | ccgatgtccaggatcaactt |
| γENaC | mouse | aacagagaaaacgccaccat | ttatgtataagatgacttgcagacca |
| Aqp-2 | mouse | tgtagaaatccgcggggacc | agggctacccaggttgtcac |
| Aqp-3 | mouse | cttttggcttcgctgtcacc | agatgggcagcttgatccag |
| Aqp-4 | mouse | ctggagccagcatgaatccag | ttcttctcttctccacggtca |
| Ca15 | mouse | gtacctggtgctacgactcc | caatgttgataggggactgggt |
| Ca2 | mouse | ctctgaccactccgcctctgctg | gtgcccttcggacccctgct |
| Ca4 | mouse | ctgctgtctttgcccctgcc | accctccctccacccccaag |
| Ca12 | mouse | accgccagtgacaagtccgaag | gggtagcaaggaggtgtagtcaggga |
| Ca13 | mouse | agagcatctttctattagcag | ggggcaaatctaggagt |
| Sglt2 | Mouse/pig/dog | ggcatgatcagccgcattct | atgagtccgcgcagaccatt |
| Tgfbr1 | Mouse/pig/dog | gacatctatgcaatgggctt | gatggatcagaaggtacaag |
| Tgfbr2 | Mouse/pig/dog | gtcatcagctgggaggacct | gagctcttgaggtccctgtg |
| Atp6v1a | mouse | cgtcagtatgatggccgact | actctgcctgctcgctcata |
| Atp6v1b1 | mouse | agctacataaccggcag | agtagaactcgtccgtca |
| Atp6v1c1 | mouse | agcattacgggtttttgt | agggcgtgagagttat |
| Atp6v1d | mouse | ggttcggtatgtgtgga | ctgcgaccatgaatgtt |
| Atp6v1e1 | mouse | cgacccgttaccaagt | tcttgcgatccccatt |
| Atp6v1f | mouse | gatcgaagacactttcagg | aggcttgacacttaaatagag |
| Atp6v1g1 | mouse | actaccgcataaacgga | acaggcgacatacact |
| Atp6v1h | mouse | actatgcgaaacgaagc | gcttctacccaggcaa |
| Atp6v0b | mouse | gacgcggtttagctca | ggaagtttccgtcagg |
| Atp6v0c | mouse | gatttgacctattcatgcgt | catccgagaacttttattgg |
| Atp6v0d2 | mouse | gccaagtgcctactcc | gcaatggcagtatcagtt |
| Atp6v0e | mouse | ccacttcgggcgttac | ccgccgagtcttgttt |
| Slc4a1 | mouse | ggctgtgctgtgggtggtgaagt | tgggagggtgggaaagtggag |
| Clcnka | mouse | gacccttcaggcgctgttcgt | ctgatgcagtgagtagctgggctg |
| Rhcg | mouse | ctggggctgacatatctgggaac | ggtgccttggaacatgggaaatac |
| Atp4a | mouse | gggtgtatcaccattctcttca | accaaccggtcacgcttag |
| Atp12a | mouse | taagatgatcccccagcaag | cccctcctttaatctccaca |
| Sglt1 | mouse | attttctgcaagagagtcaa | aagaggatgatggcaaagta |
| Vasopressin | mouse | gtgcccacctatgctcgc | cagcagatgcttggtccga |

**Supplemental table 2.** **qPCR renal pH-regulating genes collecting duct.** mRNA expression levels of V-type ATPase subunits, Anion exchanger 1, Chloride channel Ka, ammonium channel Rhcg and H^+^/K^+^-ATPases in kidneys of EMPA-treated (30 mg/kg/day; 8 weeks; n=8) and control mice (n=8). Relative expression of target genes was calculated using the 2^-ΔCt^ analysis method. The housekeeper Ct used for normalization was obtained from a pool of Ct values of different housekeeping genes, such as ß-actin, Gapdh, Rpl32 and 18s ribosomal RNA. cDNA transcription and gene-specific mRNA quantification were performed as described in the material and methods section using the SYBR Green PCR detection method. Primer sequences can be found in supplemental table 1.

| Protein | Gene | H_2_O (±SEM) | EMPA (±SEM) | p-value |
| --- | --- | --- | --- | --- |
| V-type ATPaseV1 subunit A | Atp6v1a | 1,35E-01 (±7,27E-03) | 1,42E-01 (±7,91E-03) | 0,537 |
| V-type ATPaseV1 subunit B1 | Atp6v1b1 | 1,89E-03 (±1,51E-04) | 1,50E-03 (±1,09E-04) | 0,065 |
| V-type ATPaseV1 subunit C1 | Atp6v1c1 | 7,11E-02 (±3,81E-03) | 6,97E-02 (±2,99E-03) | 0,774 |
| V-type ATPaseV1 subunit D | Atp6v1d | 3,29E-02 (±3,59E-03) | 3,48E-02 (±1,34E-03) | 0,628 |
| V-type ATPaseV1 subunit E1 | Atp6v1e1 | 3,45E-02 (±1,70E-03) | 3,03E-02 (±2,36E-03) | 0,168 |
| V-type ATPaseV1 subunit F | Atp6v1f | 2,34E-01 (±1,13E-02) | 2,38E-01 (±1,12E-02) | 0,82 |
| V-type ATPaseV1 subunit G1 | Atp6v1g1 | 1,09E-01 (±7,68E-03) | 1,06E-01 (±7,36E-03) | 0,783 |
| V-type ATPaseV1 subunit H | Atp6v1h | 5,22E-02 (±1,78E-03) | 5,66E-02 (±4,09E-03) | 0,336 |
| V-type ATPaseV0 subunit B | Atp6v0b | 4,52E-03 (±3,39E-04) | 4,66E-03 (±2,48E-04) | 0,742 |
| V-type ATPaseV0 subunit C | Atp6v0c | 5,67E-01 (±1,80E-02) | 5,47E-01 (±3,00E-02) | 0,571 |
| V-type ATPaseV0 subunit D2 | Atp6v0d2 | 1,10E-02 (±6,96E-04) | 1,15E-02 (±3,93E-04) | 0,609 |
| V-type ATPaseV0 subunit E | Atp6v0e | 1,66E-01 (±4,37E-03) | 1,69E-01 (±8,98E-03) | 0,777 |
| Anion exchanger 1 (AE1) | Slc4a1 | 1,93E-03 (±1,12E-04) | 1,64E-03 (±7,92E-05) | 0,051 |
| Chloride channel Ka | Clcnka | 4,63E-03 (±3,16E-04) | 5,65E-03 (±3,72E-04) | 0,054 |
| Mouse Rhesus blood group-associated C glycoprotein | Rhcg | 1,58E-04 (±1,35E-05) | 1,66E-04 (±1,22E-05) | 0,674 |
| ATPase H^+^/K^+^ Transporting Subunit Alpha | Atp4a | 1,40E-02 (±1,68E-03) | 1,63E-02 (±2,12E-03) | 0,395 |
| ATPase H^+^/K^+^ Transporting Non-Gastric Alpha2 Subunit | Atp12a | 1,64E-05 (±3,81E-06) | 2,09E-05 (±3,74E-06) | 0,405 |

# Supplementary Figures

**Supplemental figure 1. Glucose and creatinine measurement in urine of EMPA-treated wildtype mice.** As proof for successful inhibition of SGLT2 by empagliflozin (30 mg/kg/day) in wildtype animals (n=8 per group), urine glucose concentration was measured after 7 weeks using glucose test strips (Contour XT, Bayer AG, Leverkusen, Germany). Urine glucose excretion was massively increased in EMPA-treated mice, whereas urine creatinine concentration was significantly decreased in EMPA-treated mice, which may be indicative of glucose-induced osmotic diuresis during SGLT2 inhibition. Creatinine was measured using a colorimetric assay (BioAssay Systems, Hayward, CA, United States). Bar charts show mean values (± SEM) and asterisks indicate p<0.05. EMPA, empagliflozin.


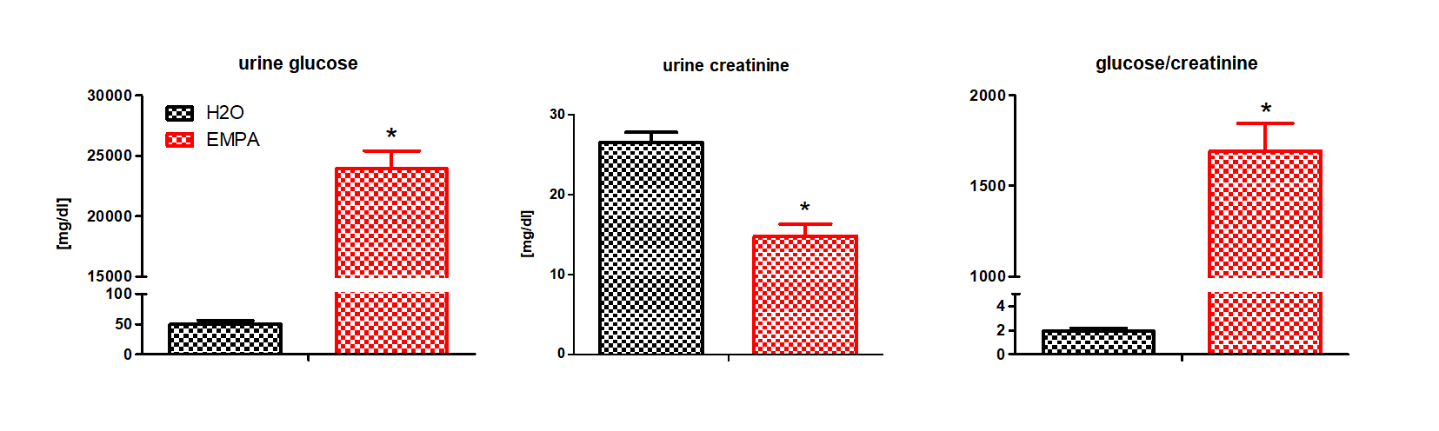


**Supplemental figure 2. Kidney wet/dry weight ratio of EMPA-treated wildtype mice.** Wildtype animals (n=10 per group) were treated with empagliflozin (30 mg/kg/day) for 2 weeks. Kidneys were removed and cut in half. One half was snap frozen for mRNA and protein while the other half was used for the measurement of the wet/dry weight ratio. Halved kidneys were weighed (wet weight) and stored in a 60 °C incubator for 3 days until kidney weight did not further decrease (dry weight). There was no difference in wet/dry weight ratio between EMPA-treated and control mice. Bar charts show mean values (± SEM). EMPA, empagliflozin.


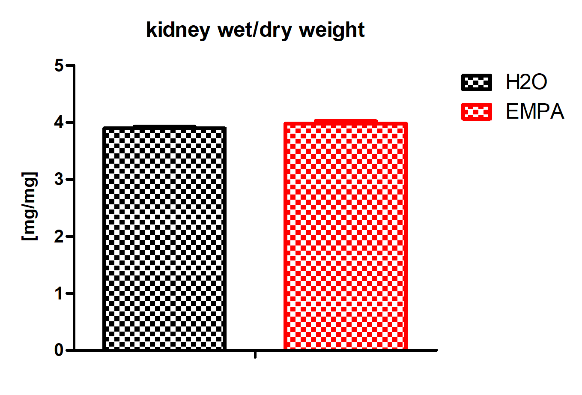


**Supplemental figure 3. Quantification of tubular cell nuclei of different nephron segments in EMPA-treated wildtype animals.** Wildtype animals were treated with empagliflozin (30 mg/kg/day) for 8 weeks and kidney sections were stained for tubular marker proteins (Megalin, Tamm-Horsfall protein, calbindin, AQP-2) as described in the material and methods section. DAPI-positive nuclei were counted for each analyzed tubule for different nephron segments. Bar charts show mean values (± SEM) and asterisks indicate p<0.05. EMPA, empagliflozin; PT, proximal tubule; LoH, loop of Henle; DCT, distal convoluted tubule; CD, collecting duct; THP, Tamm-Horsfall protein; AQP2, aquaporine-2.


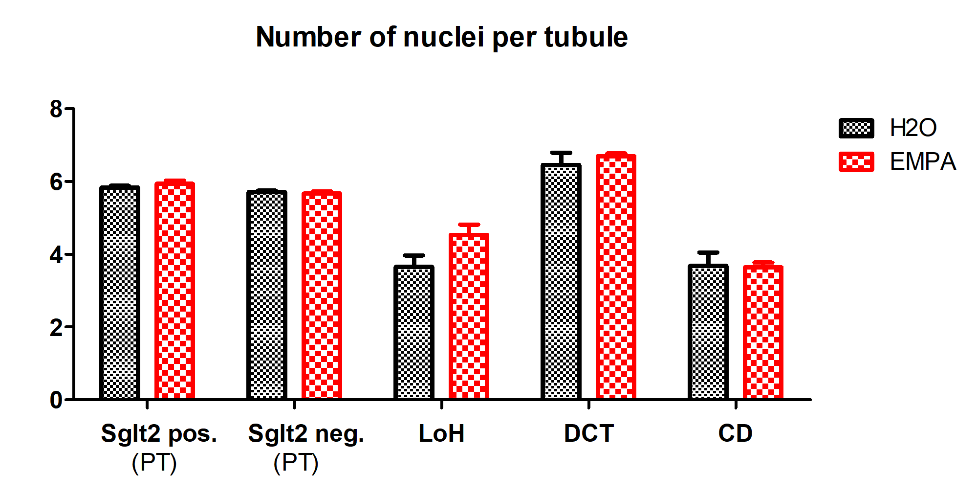


**Supplemental figure 4. mRNA quantification of Sglt2, Tgfbr1 and Tgfbr2 in renal cell lines.** LLC-PK1 cells (immortalized, porcine proximal tubule cells), MDCK-C7 cells (immortalized, canine distal tubule cells) and M-1 cells (immortalized, murine collecting duct cells) were cultivated as described in the material and methods section. mRNA was extracted from unstimulated cells using the TRIsure™ Kit (BioCat GmbH, Heidelberg, Germany). (A) qPCR measurements revealed the highest expression for Sglt2 in LLC-PK1 (n=4-5 per cell line). MDCK cells had most Tgfbr1 (B) and Tgfbr2 (C) mRNA compared to LLC-PK1 and M-1 cells (n=4-5 per cell line). Relative expression of target genes was calculated using 2^-ΔCt^ analysis method. For normalization the housekeeping gene Rpl32 was used. cDNA transcription and gene-specific mRNA quantification were performed as described in the material and methods section using the SYBR Green PCR detection method. Primer sequences can be found in supplemental table 1. Bar charts show mean values (± SEM) and asterisks indicate p<0.05.

**
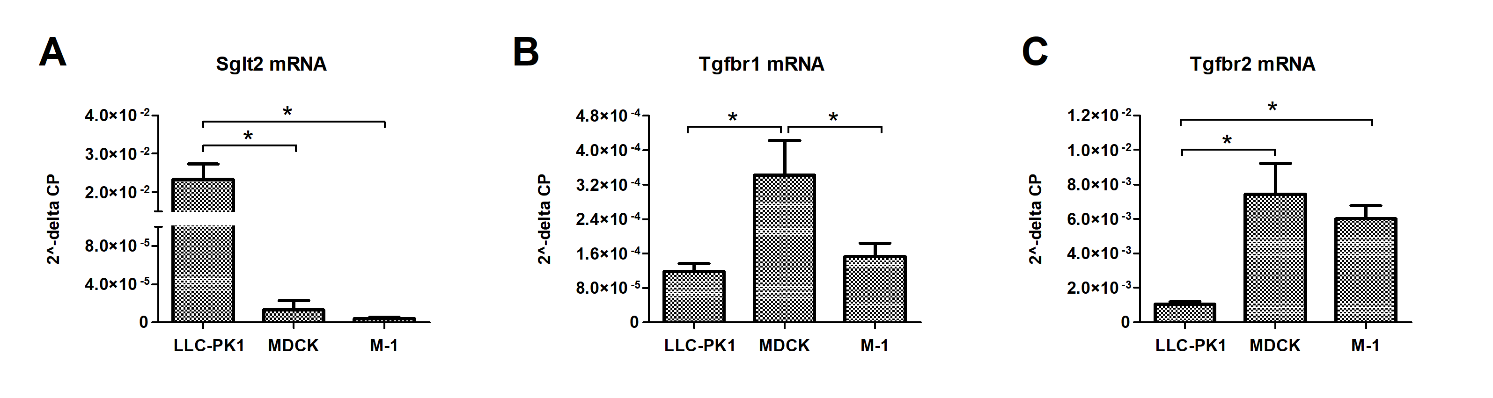
**

**Supplemental figure 5. Western blot analysis for beta-/gamma-ENaC in EMPA-treated wildtype mice.** Wildtype animals were treated with empagliflozin (30 mg/kg/day) for 8 weeks. Kidneys were lysed in RIPA buffer plus proteinase inhibitor and reducing Laemmli buffer was added to protein samples. Equal amounts of protein (50 μg per lane) were separated by a gradient 4-15 % SDS-PAGE and transferred to a PVDF membrane. After blocking with 5 % non-fat milk in TBS-tween, the membrane was incubated overnight with specific antibodies for the beta-/gamma-ENaC subunit (beta-ENaC 1:20 000; gamma-ENaC 1:40 000), kindly provided by Prof. Johannes Loffing (Institute of Anatomy, University of Zurich, Switzerland). After washing and incubation with HRP-conjugated secondary antibody (CS7074, 1:2000, Cell Signaling, Danvers, MA, United States), images were developed using luminol and visualized by a digital imaging system. For normalization, the membrane was stripped and re-incubated with an antibody against GAPDH (MA5-15738, 1:2000, Thermo Fisher Scientific GmbH, Dreieich, Germany). There was no significant difference in ENaC protein abundance between EMPA-treated and control mice. Protein bands were quantified using the Image Studio™ Lite Quantification Software (LI-COR Biotechnology; Lincoln, NE, United States). Bar charts show mean values (± SEM). EMPA, empagliflozin.


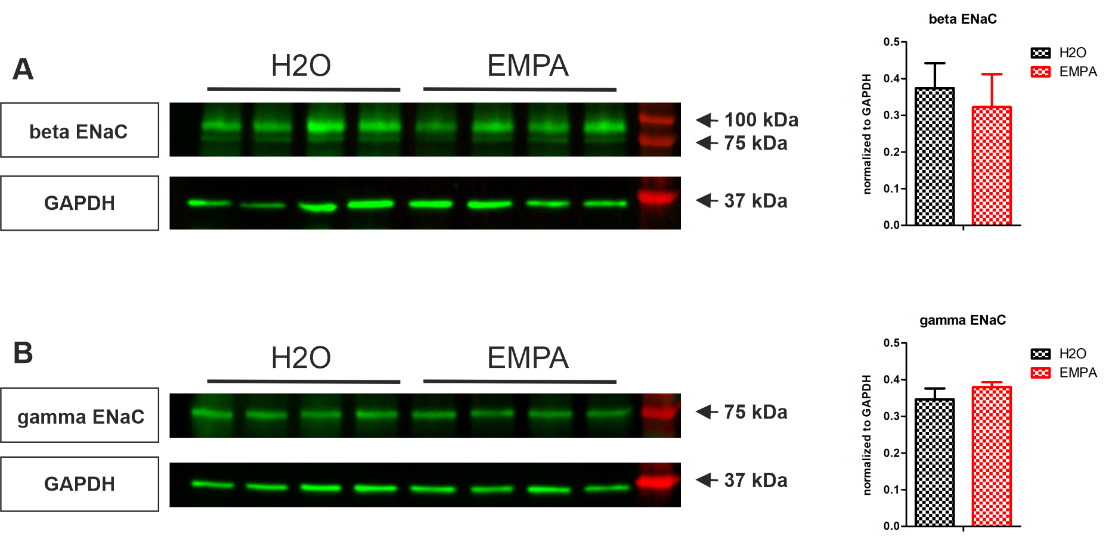


**Supplemental figure 6. GFR measurement in EMPA-treated wildtype mice.** Wildtype animals were treated with empagliflozin (30 mg/kg/day) for 8 weeks and GFR was measured using the transdermal GFR technology (MediBeacon Inc., Mannheim, Germany) described in detail in a previous study (Tauber et al., 2021). In brief, a GFR monitor was installed on the back of the animal and Fluorescein isothiocyanate (FITC)-labeled sinistrin (15 mg/100 g bodyweight) was injected intravenously. FITC-sinistrin clearance was recorded for 90 min and FITC-sinstrin t_1/2_ was converted to GFR using a mouse-specific conversion factor (Schreiber et al., 2012). EMPA-treated animals (n=8) had a similar GFR as H_2_O control animals. Bar charts show mean values (± SEM). EMPA, empagliflozin.

**
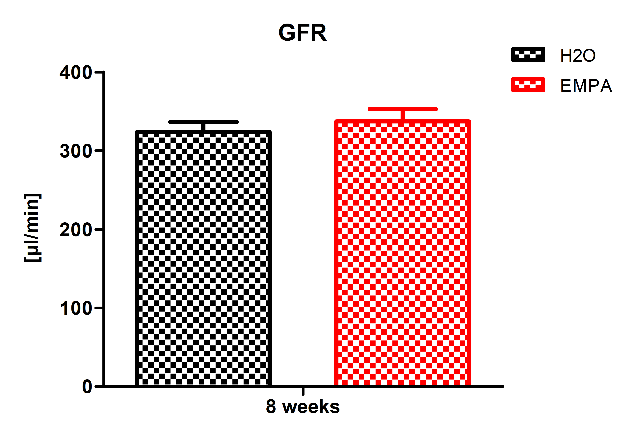
**

**Supplemental figure 7. Sglt1 mRNA expression in EMPA-treated animals.** mRNA expression level of Sglt1 was measured in kidneys of EMPA-treated (30 mg/kg/day; 8 weeks; n=8) and control mice (n=8). Relative expression was calculated using 2^-ΔCt^ analysis method. The housekeeper Ct used for normalization was obtained from a pool of Ct values of different housekeeping genes, such as ß-actin, Gapdh, Rpl32 and 18s ribosomal RNA. cDNA transcription and gene-specific mRNA quantification were performed as described in the material and methods section using the SYBR Green PCR detection method. Primer sequences can be found in supplemental table 1. Bar charts show mean values (± SEM). EMPA, empagliflozin.

**
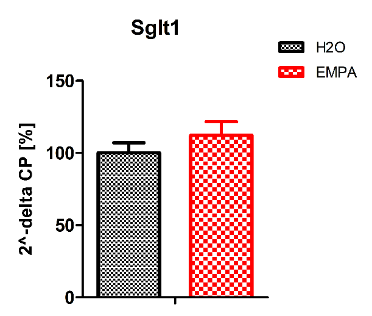
**

**Supplemental figure 8. Hif2α localization and quantification in kidneys of EMPA-treated animals.** (**A**) Overview images of Hif2α stained kidneys (brown) of untreated wildtype mice and 8 week EMPA-treated mice (30 mg/kg/d). Hif2α staining was performed following the protocol for Hif1α described in the material and methods section using a rabbit anti Hif2α antibody (NB100-122SS, 1:10 000, Novus Biologicals, Centennial, CO, United States). (**B**) Immunofluorescence co-staining of Hif2α (black) with marker proteins for the proximal tubule (megalin; red), thick ascending limb (Tamm-Horsfall protein, THP, purple), distal convoluted tubule (calbindin; orange), principle (Aqp-2; yellow) and intercalated (V-ATPase; green) cells of the collecting duct. **(C)** Western blot analysis for Hif2α (NB100-122SS, 1:1000, Novus Biologicals, Centennial, CO, United States) was performed on whole kidney lysates following the protocol described in supplemental figure 5. Renal Hif2α protein expression was not affected by EMPA treatment, shown by Hif2α staining (A) and western blot quantification (C). In contrast to Hif1α, Hif2α was expressed ubiquitously along the kidney and found in glomeruli, tubules (loop of henle, distal tubule, collecting duct) and interstitial cells. Bar charts show mean values (± SEM). EMPA, empagliflozin; THP, Tamm-Horsfall protein; Aqp-2, aquaporine-2.


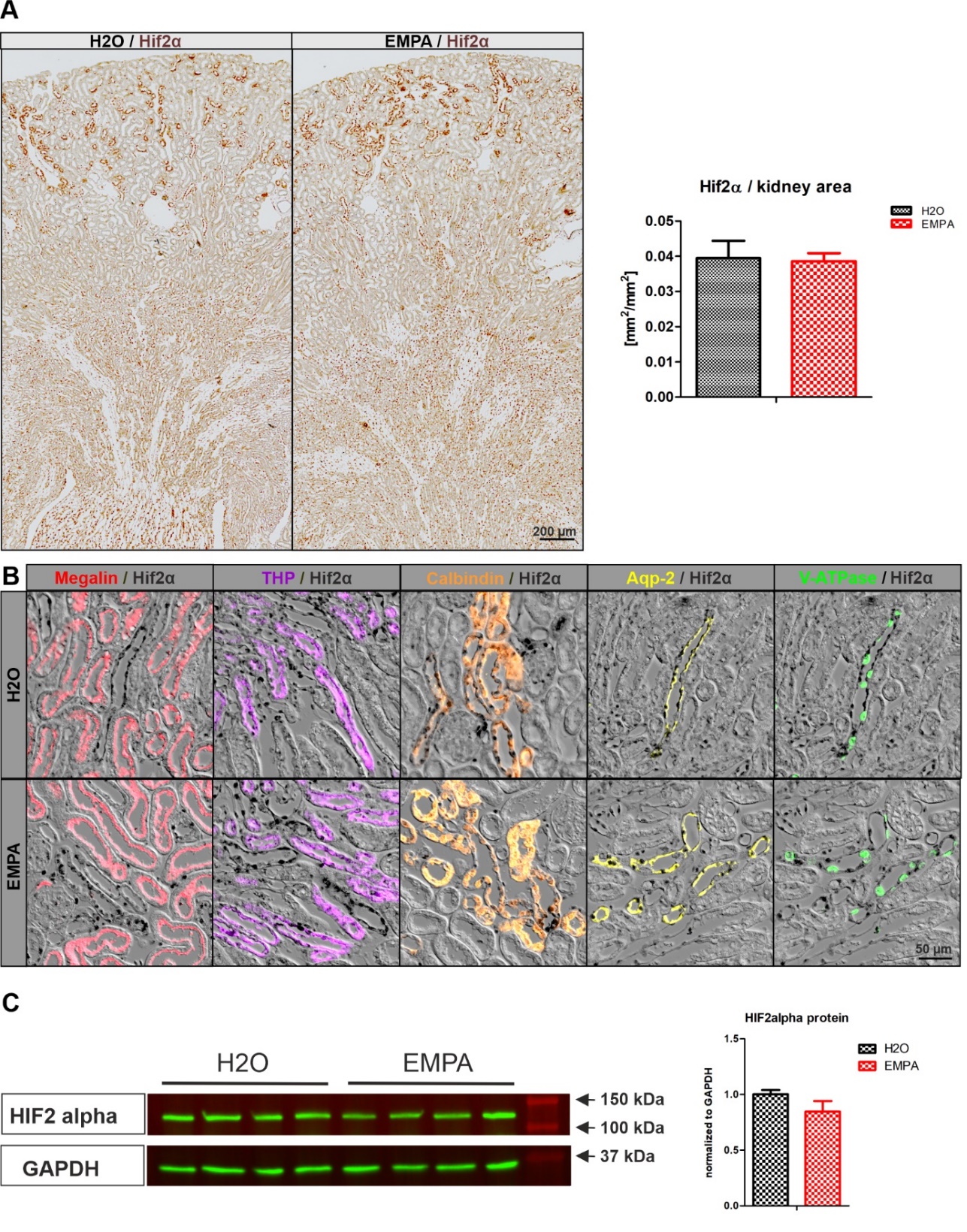


**REFERENCES (Supplements)**

Schreiber, A., Shulhevich, Y., Geraci, S., Hesser, J., Stsepankou, D., Neudecker, S., et al. (2012). Transcutaneous measurement of renal function in conscious mice. *Am J Physiol Renal Physiol* 303(5)**,** F783-788. doi: 10.1152/ajprenal.00279.2012.

Tauber, P., Sinha, F., Berger, R.S., Gronwald, W., Dettmer, K., Kuhn, M., et al. (2021). Empagliflozin Reduces Renal Hyperfiltration in Response to Uninephrectomy, but Is Not Nephroprotective in UNx/DOCA/Salt Mouse Models. *Front Pharmacol* 12**,** 761855. doi: 10.3389/fphar.2021.761855.
